# Early Initiation of Tocilizumab Treatment Against Moderate-to-Severe Myelitis in Neuromyelitis Optica Spectrum Disorder

**DOI:** 10.3389/fimmu.2021.660230

**Published:** 2021-10-21

**Authors:** Chen Du, Pei Zeng, Jin-Rui Han, Tian-Xiang Zhang, Dongmei Jia, Fu-Dong Shi, Chao Zhang

**Affiliations:** Department of Neurology, Tianjin Neurological Institute, Tianjin Medical University General Hospital, Tianjin Medical University, Tianjin, China

**Keywords:** myelitis, acute attack, tocilizumab, EDSS, annualized relapse rate

## Abstract

**Background:**

Interleukin-6 receptor blockade is effective in reducing the risk of relapses in neuromyelitis optica spectrum disorder (NMOSD). However, its efficacy during acute attacks of NMOSD remains elusive.

**Objective:**

We investigated the effects of tocilizumab on disability during acute attacks, as well as its maintenance, in patients with moderate-to-severe myelitis.

**Methods:**

Nineteen patients with NMOSD received tocilizumab treatment as add-on to high-dose methylprednisolone (HDMP) in acute myelitis and twenty-two patients who only received HDMP were compared. Disease disability was assessed using a multi-level scaling system that included the expanded disability status scale (EDSS), Hauser ambulation index (HAI), modified Rankin scale (mRS), pain numerical rating scale (NRS), functional assessment of chronic illness therapy-fatigue scale (FACIT-F), activity of daily living (ADL), EuroQol five-dimensions-three-level questionnaire (EQ-5D-3L), and sensory function score and bowel and bladder function score in Kurtzke functional systems scores (FSS).

**Results:**

Improved EDSS, HAI, and mRS, as well as increased ADL and EQ-5D-3L were significant in patients on tocilizumab compared with those on steroids as monotherapy at 3 months (*p* < 0.05). Both groups of patients showed improved pain, fatigue, sensory function, and autonomic function at follow-ups, compared with baseline respectively. The changes in NRS, FACIT-F, and sensory and autonomic FSS showed no significant differences between the two groups. Tocilizumab significantly lowered the risk of relapses (HR = 0.21, 95% CI 0.06–0.76, *p* = 0.017) and reduced the annualized relapse rate compared with those by steroids (0.1 ± 0.2 *vs* 0.5 ± 0.6, *p* = 0.013).

**Conclusion:**

Early initiation of tocilizumab provided a safe and effective add-on alternative during attacks, and its maintenance contributed to a significant reduction of relapse rate in NMOSD.

## Introduction

Neuromyelitis optica spectrum disorder (NMOSD) is a relapsing autoimmune inflammatory disease of the central nervous system, characterized by attacks of transverse myelitis, optic neuritis, refractory hiccups, and other features of the nervous system (1–3). The symptoms of myelitis are usually severe and affect the quality of life of the patients (4–6). Early effective treatments during acute attacks are necessary to reduce the risk of an increased irreversible neurologic disability. High-dose methylprednisolone (HDMP; 1000 mg/d for five consecutive days) is considered the first-line standard care in acute attacks (7). Intravenous immunoglobulin (IVIG), plasma exchange, or apheresis also show benefits as add-on treatments in patients who are unresponsive to HDMP (8–11). However, patients can still commonly experience sequelae despite aggressive immunosuppression (12).

The role of IL-6 in the immunopathogenesis of acute attacks in patients with NMOSD has recently been proposed. Sera and Cerebro-Spinal Fluid (CSF) IL-6 levels were significantly higher during attacks of myelitis in patients with NMOSD than in patients with multiple sclerosis and correlated with the expanded disability status scale (EDSS) (13, 14). In addition, CSF IL-6 levels correlated with those of AQP4-IgG and glial fibrillary acidic protein, a marker of astrocyte damage (15). Pathogenic IL-6 may also drive disease activity in NMOSD by promoting B-cell differentiation into antibody-secreting cells, reducing blood–brain barrier integrity, and activating proinflammatory Th17 cells (16–18). By inhibiting the IL-6 signaling pathway with IL-6 receptor monoclonal antibody, satralizumab or tocilizumab significantly reduced the risk of relapses during periods of remission of NMOSD (3, 19, 20). However, it remains uncertain whether IL-6R inhibition is also highly effective during acute attacks.

In this retrospective study, we aimed to investigate whether the early initiation of tocilizumab treatment as an add-on during acute attacks of moderate-to-severe myelitis, followed by regular infusions, reduced the severity of disability and sustained long-term efficacy by improving symptoms.

## Methods

### Patients

We identified patients with confirmed NMOSD, according to the 2015 international consensus diagnostic criteria (21). All patients were admitted to the Department of Neurology, Tianjin Medical University General Hospital, between June 2017 and July 2020. The criteria for inclusion were that 1) patients were experiencing acute attacks, where 2) a moderate-to-severe myelitis attack was defined as having any one of the following: EDSS ≥ 3.5 for the first-ever myelitis (22) at least a two-point increase of the EDSS score if the previous score was ≤5.0, or at least a one-point worsening of their EDSS score if the previous score was ≥5.5. The exclusion criteria were the patients who (1) used B-cell depleting agents and complement inhibitors during attacks and remission, (2) initiated tocilizumab therapy after acute attacks, and (3) lost follow-ups (4) concomitant with other diseases that may affect disability evaluation (e.g., stroke and Parkinson’s disease). The study was approved by the Institutional Review Board of Tianjin Medical University General Hospital.

### Treatment, Clinical Evaluation, and Outcomes

HDMP or HDMP+IVIG was administered to patients to treat acute attacks. In the tocilizumab group, treatment with tocilizumab was initiated at the dose of 8 mg/kg within 2 weeks of the attack onset, followed by a routine infusion at 4-week intervals. Prednisone was gradually tapered to 10–15 mg/day for maintenance. In the prednisone group, prednisone was gradually tapered to a dose of 10–15 mg/day for attack prevention (**Supplementary Figure 1**).

We evaluated the disability of patients using a set of clinical scales, including the EDSS, Hauser ambulation index (HAI), modified Rankin scale (mRS), functional assessment of chronic illness therapy-fatigue scale (FACIT-F), numerical rating scale (NRS), activity of daily living (ADL) score, EuroQol five-dimensions-three-level questionnaire (EQ-5D-3L), and sensory function score and bowel and bladder function score in Kurtzke functional systems scores (FSS). The scores were recorded and calculated retrospectively from available records for the following time periods: discontinuation of HDMP or HDMP+IVIG (baseline) at 1, 3, 6, and 12 months and on follow-ups (when available).

The primary outcome was an improvement of the EDSS score at each time point. The secondary outcomes included score changes from the baseline to the follow-up time points on the following assessments: HAI, mRS, FACIT-F, NRS, ADL score, EQ-5D-3L, and sensory function score and bowel and bladder function score in FSS. An additional secondary outcome was the annualized relapse rate (ARR), calculated as the total number of attacks per patient, divided by the total observation time in years. Attacks during follow-ups were defined as new or worsening objective neurologic symptoms attributable to NMOSD, that lasted for at least 24 h and occurred more than 30 days after the previous attack (19, 20).

Adverse events (AEs) were recorded according to the National Cancer Institute Common Terminology Criteria for Adverse Events (NCI CTCAE) version 5.0.

### Statistical Analysis

We performed statistical analyses using the Stata version 16 software (StataCrop LP, College Station, TX, USA). Data are presented by *n* (%), mean (SD), or median (IQR). Categorical variables were compared using the chi-squared test. Generalized estimating equation (GEE) for within-participant correlations of repeated outcome measures and adjusts for potential confounding variables. Inter- and intra-group data difference between the baseline and follow-ups were compared at different time points by using multivariate analysis of covariance (MANCOVA) to avoid confounding factors. Survival analysis of relapse-free in two groups were performed by the Kaplan–Meier method. Stratified Cox proportional hazards regressions were conducted for subgroup analysis based on sex, age of onset, disease duration, presence of AQP4-ab in serum and number of previous attacks. *P* values < 0.05 were considered significant.

## Results

### Demographics and Baseline Clinical Characteristics

**Figure 1:**
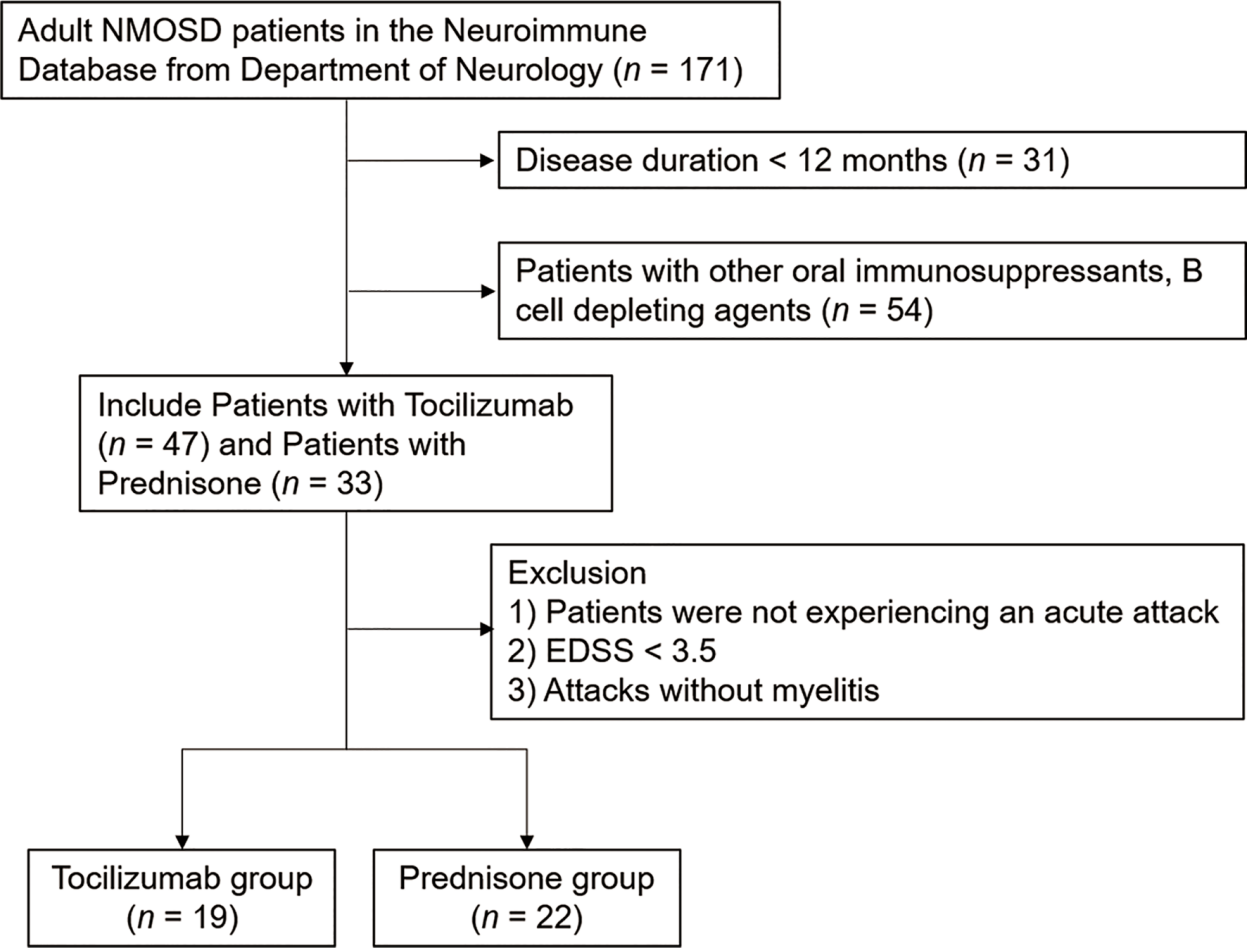
Flowchart of the patients enrolled in the study. The patients were retrospectively enrolled between June 1, 2017 and July 1, 2020. All patients were admitted to the Department of Neurology, Tianjin Medical University General Hospital.

After excluding 130 patients who did not meet the inclusive criteria from the database, we identified 41 patients who were experiencing acute attacks. Of these, 19 (46.34%) received tocilizumab as an add-on treatment, and 22 (53.66%) received steroids (prednisone) as the only treatment (**Figure 1**). All patients experienced longitudinally extensive transverse myelitis. The mean age at the most recent acute attack was 44.74 ± 15.83 years in the tocilizumab group and 52.68 ± 15.74 years in the prednisone group. Twenty-seven (65.85%) patients were AQP4-IgG seropositive. Two (10.53%) patients from the tocilizumab group and three (13.64%) patients from the prednisone group also presented with optic neuritis. Ten (52.63%) patients from the tocilizumab group and eight (36.36%) in the prednisone group had symptomatic cerebral syndrome. Tocilizumab treatment was initiated at 10.05 ± 4.93 days after the onset date of acute attacks. Demographic characteristics are listed in **Table 1**.

**Table 1:** Demographics and baseline clinical characteristics of patients with longitudinally extensive transverse myelitis (LETM) in neuromyelitis optica spectrum disorder (NMOSD).

	TCZ	Prednisone	*p* value
Number of patients(N)	19	22	/
Age at onset (year, mean ± SD)	44.74 ± 15.83	52.68 ± 15.74	0.077
Sex (F/total, percent)	16/19 (84.21%)	17/22 (77.27%)	/
Anti-AQP4 (positive, percent)	12/19 (63.16%)	15/22 (68.18%)	/
Treatment in acute attacks			
HDMP (≥ 500mg/day)	17 (89.47%)	20 (90.91%)	/
IVIG (0.4g/kg/d×5d)	1 (5.26%)	1 (4.55%)	/
HDMP+IVIG	1 (5.26%)	1 (4.55%)	/
Number of previous attacks (median, IQR)	2.0 (1.0-3.5)	3.0 (2.0-4.8)	0.073
Annualized relapse rate (mean ± SD)	1.18 ± 0.96	1.07 ± 1.74	0.872
Follow-up time (years, mean ± SD)	1.33 ± 0.63	1.10 ± 0.51	0.116
Concurrent involvement			
Optic neuritis	2 (10.53%)	3 (13.64%)	
Symptomatic cerebral syndrome	10 (52.63%)	8 (36.36%)	
Acute brainstem syndrome	1 (5.26%)	1 (4.55%)	
Score at onset of the acute attack			
EDSS (median, IQR)	7.0 (3.5-8.0)	7.0 (3.1-8.0)	0.979
HAI (median, IQR)	7.0 (1.0-8.0)	7.0 (1.0-9.0)	0.592
mRS (median, IQR)	4.0 (0.0-5.0)	4.0 (1.0-5.0)	0.703
Pain (median, IQR)	6.0 (3.5-7.0)	7.0 (2.8-8.0)	0.504
FACIT-F (median, IQR)	43.0 (16.0-47.0)	41.5 (30.3-45.8)	0.958
ADL (median, IQR)	25.0 (20.0-95.0)	25.0 (15.0-90.0)	0.700
EQ-5D-3L (median, IQR)	0.26 (0.16-0.73)	0.20 (0.11-0.77)	0.580
FSS-Sensory function (median, IQR)	2.0 (2.0-3.0)	3.0 (2.0-3.0)	0.314
FSS-Bowel and bladder function (median, IQR)	1.0 (0.50-3.0)	1.0 (1.0-2.8)	0.914

SD, Standard deviation; IQR, interquartile range.

### EDSS, HAI, and mRS

**Figure 2:**
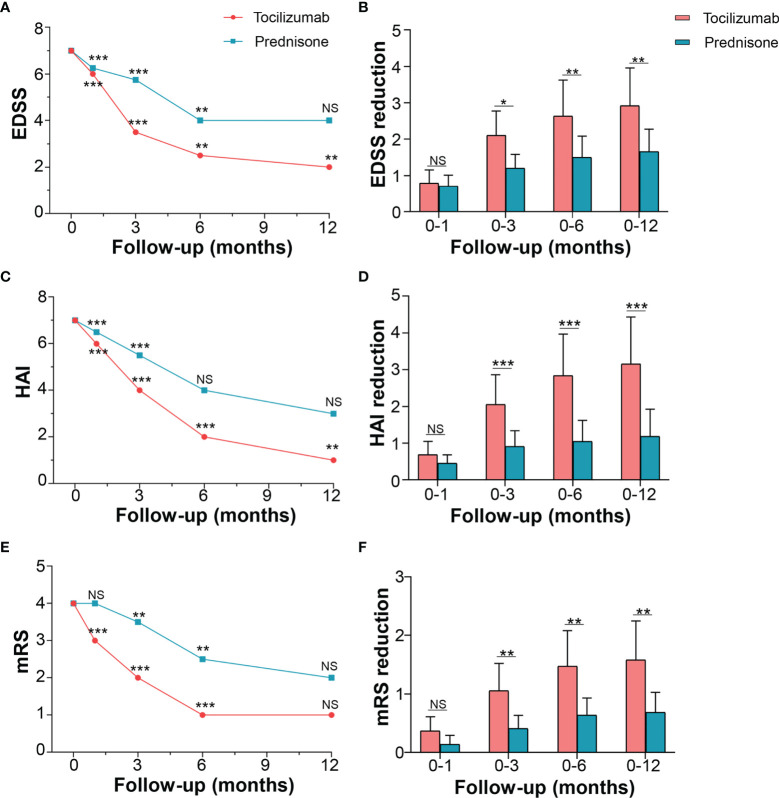
Effects of tocilizumab and prednisone on motor function. **(A)** EDSS changes from the baseline to the one-year follow-up in the tocilizumab and prednisone groups. **(B)** Comparison of EDSS reduction rate between the wo groups. **(C)** HAI changes and **(D)** comparison between the two groups. **(E)** mRS changes and **(F)** comparison between the two groups. EDSS: expanded disability status scale; HAI: Hauser ambulation index; mRS: modified Rankin scale. * represents a significant decline in score between the current and previous follow-ups. *p < 0.05; **p < 0.01; ***p < 0.001; NS, not significant.

For the primary outcome, tocilizumab reduced the median EDSS scores from a baseline (i.e., at the time of attack onset) of 7.0 (IQR 3.5–8.0) to a median of 6.0 (IQR 2.5–7.0, *p* < 0.001) at 1 month, 3.5 (IQR 2.0–5.75, *p* < 0.001) at 3 months, and 2.5 (IQR 2.0–5.5, *p* = 0.003) at 6 months. At the 12-month follow-up, EDSS scores of patients treated with tocilizumab declined to a median of 2.0 (IQR 2.0–5.0, *p* < 0.001). EDSS scores of patients from the prednisone group declined from a median of 7.0 (IQR 3.1–8.0) to a median of 6.3 (IQR 2.5–7.4, *p* < 0.001) at 1 month, 5.8 (IQR 2.0–6.9, *p* < 0.001) at 3 months, 4.0 (IQR 2.0–6.5, *p* = 0.003) at 6 months, and 4.0 (IQR 2.0–6.5, *p* = 0.109) at 12 months (**Figure 2A**), indicating no further reduction in EDSS score between the 6-month and 12-month periods. Inter-group adjusted analysis showed that tocilizumab significantly reduced EDSS scores at 3 months [median difference 1.5 (IQR (1.5–3.0) *vs* 1.0 (IQR 0.6–1.5), *p* = 0.010] compared to those with prednisone and that the effect was maintained until the 12-month assessment [2.0 (IQR 1.5–4.8) *vs* 1.0 (IQR 1.0–2.0), *p* = 0.003] (**Figure 2B**).

The motor function was also evaluated using HAI and mRS. The HAI score for patients in the tocilizumab group decreased from a baseline median of 7.0 (IQR 1.0–8.0) to a median of 6.0 (IQR 1.0–7.5, *p* < 0.001) at 1 month, 4.0 (IQR 0.0–5.0, *p* < 0.001) at 3 months, 2 (IQR 0.0–4.0, *p* < 0.001) at 6 months, and 1.0 (IQR 0.0–3.0, *p* = 0.009) at 12 months. The changing HAI scores for patients on prednisone followed a similar trend (**Figure 2C**). However, compared with prednisone, tocilizumab produced a significant reduction in HAI score at 3 months, which persisted over the following 9 months (**Figure 2D**). In mRS, both groups of patients improved at 3 months and 6 months (**Figure 2E**) compared with baseline. A significant reduction rate in mRS was seen in the tocilizumab group compared to that in the prednisone group, at 3, 6, and 12 months (*p* < 0.05) (**Figure 2F**).

### Pain NRS and FACIT-F Scale

**Figure 3:**
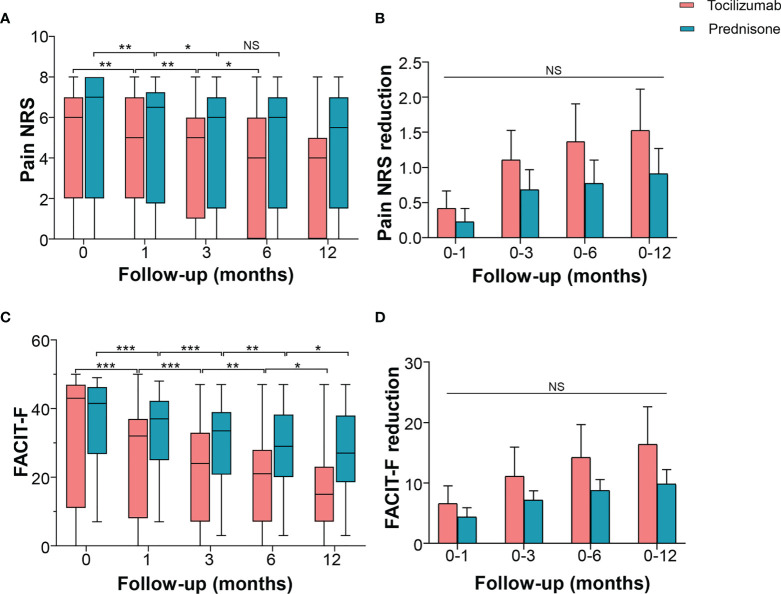
Effects of tocilizumab and prednisone on pain and fatigue. **(A)** Pain NRS changes at baseline, 1 month, 3 months, 6 months, and 12 months in patients receiving tocilizumab and prednisone. **(B)** Pain NRS reduction rate was compared between the two groups. **(C)** FACIT-F score changes at the baseline, 1 month, 3 months, 6 months, and 12 months in patients receiving tocilizumab and prednisone. **(D)** Comparisons of FACIT-F reduction rate between the two groups. FACIT-F: Functional Assessment of Chronic Illness Therapy-Fatigue Scale; NRS: numerical rating scale. *p < 0.05; **p < 0.01; ***p < 0.001; NS, not significant.

Changes in pain and fatigue levels in patients with NMOSD were evaluated using NRS and FACIT-F, respectively. The NRS score in the tocilizumab group decreased to a median of 5.0 (IQR 3.0–7.0, *p* = 0.002) at 1 month, 5.0 (IQR 2.0–5.0, *p* = 0.009) at 3 months, and 4.0 (IQR 1.5–5.5, *p* = 0.013) at 6 months, with no further decline observed after 6 months. In the prednisone group, pain NRS decreased to a median of 6.5 (IQR 2.5–7.0, *p* = 0.008) at 1 month and 6.0 (IQR 2.5–7.0, *p* = 0.013) at 3 months, with no further reduction detected after 3 months (**Figure 3A**). Inter-group multivariate analysis revealed no significant difference in the reduced NRS between the two groups at the corresponding time points (**Figure 3B**).

In the tocilizumab group, the FACIT-F score reduced from a baseline median of 43 (IQR 16–47) to a median of 32.0 (IQR 12.5–36.5), *p* < 0.001) at 1 month, 24.0 (IQR 9.0–32.5, *p* = 0.002) at 3 months, 21.0 (IQR 7.0–26.0, *p* = 0.004) at 6 months, and 15.0 (IQR 7.0–22.0, *p* = 0.014) at 12 months. In the prednisone group, the FACIT-F score decreased from a baseline median of 41.5 (IQR 30.3–45.8) to a median of 37.0 (IQR 27.0–42.0, *p* < 0.001) at 1 month, 33.5 (IQR 23.8–39.0, *p* < 0.001) at 3 months, 29.0 (IQR 23.8–38.0, *p* = 0.004) at 6 months, and 27.0 (IQR 21.0–37.8, *p* = 0.025) at 12 months (**Figure 3C**). However, these changes in the FACIT-F score showed no difference between the two groups (**Figure 3D**). Compared with prednisone, tocilizumab did not exhibit a higher efficacy as an add-on to improve pain and fatigue.

### Quality of Life

**Figure 4:**
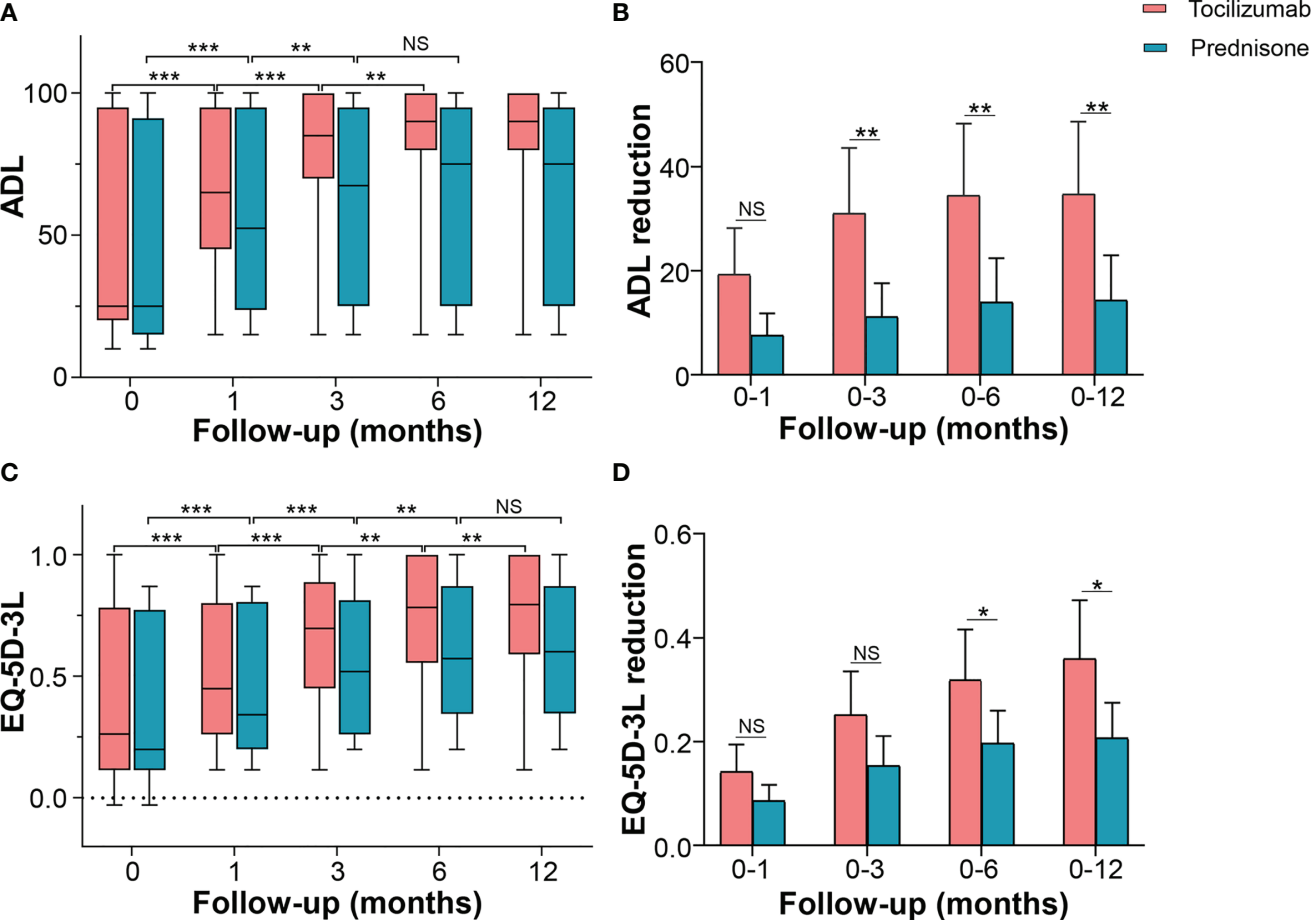
Effects of tocilizumab and prednisone on quality of life. **(A)** Changes of ADL score at baseline, 1 month, 3 months, 6 months, and 12 months in patients receiving tocilizumab and prednisone. **(B)** Comparisons of ADL improvements between the two groups. **(C)** Changes in EQ-5D-3L score at baseline, 1 month, 3 months, 6 months, and 12 months in patients receiving tocilizumab and prednisone. **(D)** Comparisons of EQ-5D-3L improvements between the two groups. ADL: activity of daily living; EQ-5D-3L: EuroQol Five-dimensions Three-level questionnaire. *p < 0.05; **p < 0.01; ***p < 0.001; NS, not significant.

ADL was used to determine self-care ability in patients with NMOSD. Tocilizumab significantly improved ADL from a baseline median of 25.0 (IQR 20.0–95.0), to a median of 65.0 (IQR 45.0–95.0, *p* < 0.001) at 1 month, 85.0 (IQR 72.5–100, *p* < 0.001) at 3 months, and 90.0 (IQR 80.0–100, *p* = 0.002) at 6 months, with no further decline from the six-month follow-up (*p* = 0.271). In the prednisone group, ADL improved from a baseline median of 25.0 (IQR 15.0–90.0) to a median of 52.5 (IQR 25.0–95.0, *p* < 0.001) at 1 month, 67.5 (IQR 25.0–95.0, *p* = 0.006) at 3 months, and with no further reduction thereafter. Inter-group multivariate analysis revealed no significant difference in the changes in improved ADL at 1 month from the baseline [10.0 (IQR 2.5–35.0) *vs* 5.0 (IQR 5.0–8.0), *p* = 0.083]. However, compared with patients on prednisone, in patients on tocilizumab, significant improvements were seen at 3 months [median difference 35.0 (IQR 5.0–52.5) *vs* 5.0 (IQR 5.0–10.0), *p* = 0.002], 6 months [median difference 35.0 (IQR 5.0–65.0) *vs* 5.0 (IQR 5.0–10.0), *p* = 0.003], and 12 months [median difference 35.0 (IQR 5.0–65.0) *vs* 5.0 (IQR 5.0–10.0), *p* =0.003] (**Figures 4A, B**).

EQ-5D-3L increased from a baseline median of 0.260 (IQR 0.157–0.733) to a median of 0.450 (IQR 0.313–0.792, *p* < 0.001) at 1 month following the initiation of tocilizumab treatment. It progressively and markedly improved to a median of 0.696 (IQR 0.460–0.841, *p* < 0.001) at 3 months, 0.783 (IQR 0.573–0.944, *p* = 0.004) at 6 months, and 0.795 (IQR 0.644–1.000, *p* = 0.009) at 12 months. In patients treated with prednisone, EQ-5D-3L showed a similar trend until the 6-month follow-up, with no further improvements detected (**Figure 4C**). Inter-group multivariate comparison analysis revealed that patients on tocilizumab showed a significant improvement in EQ-5D-3L compared with that in patients on prednisone, at 3 months [median difference 0.250 (IQR 0.126–0.349) *vs* 0.135 (IQR 0.086–0.214), *p* = 0.044], 6 months [median difference 0.338 (IQR 0.135–0.467) *vs* 0.198 (IQR 0.092–0.246), *p* = 0.025], and 12 months [median difference 0.373(IQR 0.135–0.514) *vs* 0.224 (IQR 0.092–0.250), *p* = 0.010] (**Figure 4D**).

### Sensory and Autonomic (Bowel and Bladder) Function

**Figure 5:**
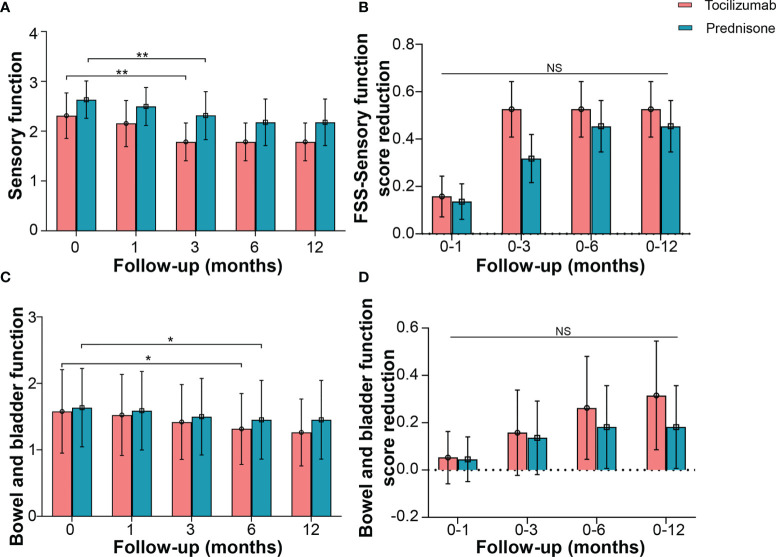
Effects of tocilizumab and prednisone on sensory function and autonomic (bowel and bladder) function. **(A)** Changes of FSS sensory function score at baseline, 1 month, 3 months, 6 months, and 12 months in patients receiving tocilizumab and prednisone. **(B)** Comparisons of FSS sensory function score reduction rate between the two groups. **(C)** Changes of FSS bowel and bladder function score in patients with neuromyelitis optica spectrum disorder (NMOSD). **(D)** Comparisons of FSS bowel and bladder function score reduction between the two groups. *p < 0.05; **p < 0.01; NS, not significant.

FSS was used to evaluate sensory and autonomic (bowel and bladder) function. Tocilizumab marginally reduced the sensory function score from a baseline median of 2.0 (IQR 2.0–3.0) to a median of 2.0 (IQR 2.0–2.0) at 3 months (*p* = 0.001). However, no significant decline was detected at the subsequent time points (**Figure 5A**). In the prednisone group, scores for sensory function showed no reduction at 1 month, compared with the baseline [median 3.0 (IQR 2.0–3.0) *vs* 3.0 (IQR 2.0–3.0), *p* = 0.062]. Significant differences were detected until the 6-month mark [median 2.0 (IQR 1.0–3.0), *p* = 0.001], and no further decline was observed at subsequent time points (**Figure 5B**).

Changes in bowel and bladder function showed a similar trend. FSS in patients treated with tocilizumab reduced from a baseline median of 1.0 (IQR 0.5–3.0) to a median of 1.0 (IQR 0.5–2.0) at 6 months (*p* = 0.031). Patients treated with prednisone also exhibited a decline in FSS at 6 months [median 1.0 (IQR 0.0–1.0), *p* = 0.019] (**Figure 5C**). No significant differences in the reduced FSS were found between the two groups at the corresponding time points (**Figure 5D**).

### Comparison of Efficacy Between AQP4-ab Positive and AQP4-ab Negative Patients

The efficacy of tocilizumab in patients with AQP4-ab (+) and AQP4-ab (-) patients are shown in **Supplementary Tables S1** and **S2**, respectively. There were no significant differences in disability index at between the two groups at baseline. After 12-month follow up, the patients showed no significant differences in disability severity (including EDSS, HAI, mRS, Pain NRS, FACIT-F, ADL, EQ-5D-3L, sensory function, and bowel and bladder function) between the two groups (*p* > 0.05, **Supplementary Table S3** and **Supplementary Figure S1**).

Inter-group covariance analysis suggested that AQP4-ab (+) patients with tocilizumab had better response in EDSS, HAI, mRS, pain NRS, FACIT-F, ADL, and EQ-5D-3L, compared with those with prednisone at 12-month follow-up. However, in AQP4-ab (-) patients, we did not find any significant differences in all disability index between the tocilizumab group and the prednisone group at 12-month follow-up (**Supplementary Table S4**).

### Relapses and Survival Analysis

**Figure 6:**
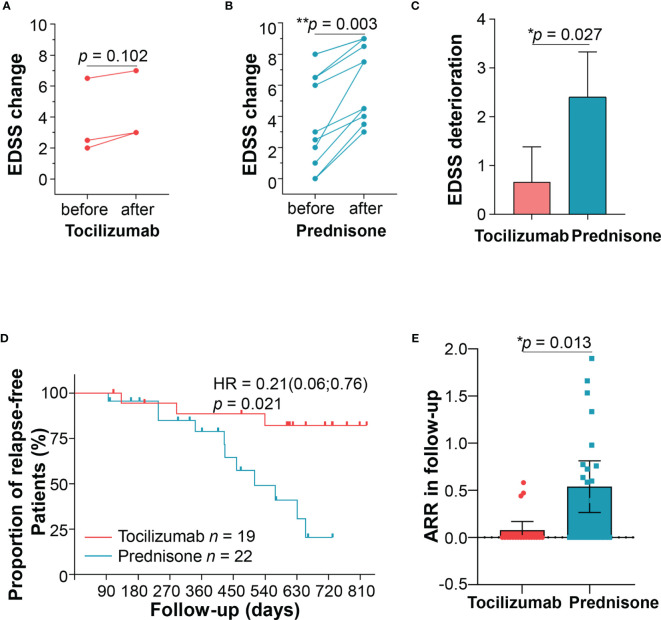
Patient relapses. Three patients relapsed in the tocilizumab group, and expanded disability status scale (EDSS) scores were compared before and after the relapse. **(B)** Eleven patients relapsed in the prednisone group, and EDSS scores were compared before and after the relapse. **(C)** Comparisons of EDSS changes between the two groups. **(D)** Kaplan–Meier curve estimating the proportion of patients who experienced no relapses during two years of follow-up in the two groups. **(E)** ARR after tocilizumab or prednisone treatment during follow-ups. ARR, annualized relapse rate.

Three patients (15.79%) in the tocilizumab group relapsed during follow-ups (**Figure 6A**). One patient had a relapse with brainstem involvement 1 year later, after prolonging the tocilizumab infusion interval over 2 months, owing to circumstances arising from the COVID-19 pandemic. Another patient, following discharge due to hyperglycemia, quickly withdrew oral corticosteroids, and had right optic neuritis 3 months later. The third patient relapsed 5 months after the initiation of tocilizumab treatment and experienced numbness and weakness in both lower limbs; MRI results showed longitudinal myelitis (T2–T8).

Eleven (50.0%) patients in the prednisone group relapsed (**Figure 6B**). Seven patients experienced disability in walking, two presented numbness of limbs, one had optic neuritis, and one developed a stubborn hiccup. Among the patients experiencing new attacks, those treated with tocilizumab showed lower EDSS increment than those treated with prednisone after adjusting confounding factors (1.13 ± 0.63 *vs* 2.40 ± 1.38, *p* = 0.027) (**Figure 6C**). Other parameters including HAI, mRS were also compared before and after relapses, as shown in **Supplementary Table S5** and **Supplementary Figure S2**. Generally, patients with prednisone tended to show a worse disability at new attacks compared with those in the tocilizumab group.

Compared with prednisone, tocilizumab showed significant reduction in the risk of relapses (HR = 0.21, 95% CI, 0.06–0.76, *p* = 0.017) (**Figure 6D**). Although both groups of patients displayed reduced ARR, the reduction was more significant in the tocilizumab group (from 1.18 ± 0.96 to 0.10 ± 0.20) than in the prednisone group (from 1.07 ± 1.74 to 0.54 ± 0.62) (*p* = 0.013; **Figure 6E**). We further performed stratified cox proportional hazards regressions for subgroup analysis based on sex, age of onset, disease duration, presence of AQP4-ab, number of previous attacks and time to initiation of tocilizumab. The results showed no significant difference at all the above index in reducing relapse risks (**Supplementary Figure S3**).

### Safety Considerations

AEs were recorded for patients who received tocilizumab (**Table 2**). We detected no severe infection during the follow-ups. The most common AE was hepatotoxicity (*n* = 10, Grade 1). Sixteen patients experienced hypoproteinemia after two cycles of tocilizumab. Laboratory examinations revealed anemia in 11 patients (1 with Grade 2 and 10 with Grade 1). Hyperlipidemia (Grade 1) occurred in nine patients. Headache (*n* = 1, Grade 1), nausea (*n* = 1, Grade 1), increased D-dimers (*n* = 3), and urinary tract infections (*n* = 1, Grade 3) were also recorded. No patients experienced AEs of Grades > 3. We recorded no serious side effects that resulted in the discontinuation of tocilizumab treatment.

**Table 2:** Adverse events during tocilizumab treatment in patients with neuromyelitis optica spectrum disorder (NMOSD).

	AQP4-Ab positive	AQP4-Ab negative
	n = 12	n = 7
Death	0	0
PMLs	0	0
Severity and type of adverse events		
Grade 1		
Hepatotoxicity	6 (50.0%)	4 (57.1%)
Hypoproteinemia	8 (66.7%)	7 (100%)
Anemia	5 (41.7%)	5 (71.4%)
Hyperlipidemia	5 (41.7%)	4 (57.1%)
Nausea	1 (8.3%)	0
Headache	1 (8.3%)	1 (14.3%)
High D-dimer level	1 (8.3%)	2 (28.6%)
Grade 2		
Anemia	0	1 (14.3%)
Hypoproteinemia	1 (8.3%)	0
Grade 3		
Infections	1 (8.3%)	0
Grade 4	0	0

PML, Progressive multifocal leukoencephalopathy; AQP4-Ab, aquaporin-4 antibody.

## Discussion

Few studies have shown the efficacy of disease-modifying drugs in acute attacks of NMOSD. Our study is the first to explore the effects of early initiation of tocilizumab in acute attacks on the disability and quality of life of patients with moderate-to-severe myelitis in NMOSD.

This study showed that the early combination of tocilizumab with high-dose steroid treatment significantly improved disability, compared with that by treatment with steroids only, in patients experiencing acute attacks of myelitis. Contrary to prior studies of IL-6R inhibition in NMOSD, wherein treatment with the IL-6R monoclonal antibody satralizumab was started after 30 days of acute attacks, tocilizumab treatment was initiated within 2 weeks of attack onset in the present study. A remarkable improvement in disability was observed at 3 months (i.e., after 3 cycles of tocilizumab). Moreover, tocilizumab was well-tolerated and highly effective at preventing attacks.

We used multiple scales to evaluate ambulatory motor function with tocilizumab treatment and compared the results with those obtained from treatment with steroids only. Specifically, the early use of tocilizumab was associated with improved ambulatory motor function, as shown from the assessment of EDSS, HAI, and mRS. However, treatment using only steroids also contributed, to some extent, to improved EDSS, HAI, and mRS scores. This indicates that tocilizumab may be effective when used early, in cases of severe disability or therapy-resistant active NMOSD, by reducing EDSS scores (23).

Intractable neuropathic pain and fatigue are two characteristic clinical symptoms in NMOSD (24). Fatigue might be correlated with a change in DAS28e and in pain (25). Consistent with previous case studies that showed improved pain and fatigue in patients with NMOSD (26), our study suggested that both neuropathic pain and fatigue during attacks could be improved with tocilizumab as an add-on or with steroids only. We detected no significant difference in the reduction of pain NRS or FACIT-F scores when comparing the two groups of patients. Similarly, in the SakuraSky trial, IL-6R inhibition with satralizumab showed no difference in neurologic pain and fatigue between the baseline and the endpoint of the study (19). As an add-on, tocilizumab showed no superiority to steroids, except in reducing the severity of pain and fatigue. Animal models showed that spinal IL-6 may mediate the mechanism underlying chronic pain (27). Although we observed a slow decrease in NRS during the period of remission, there was no difference between 1 month and 6 months following the attacks. Further studies involving larger samples of patients receiving tocilizumab are required to determine whether IL-6R inhibition would contribute to reducing pain during long-term infusions.

NMOSD negatively affects the quality of life of patients, their families, and their social network (4). We provide evidence that the early use of tocilizumab as an add-on during attacks accelerated the improvement of the self-care ability and health status compared with those in patients who received steroids only. These findings mirrored the efficacy of tocilizumab in rheumatoid arthritis using EQ-5D (28). Bladder and bowel dysfunction is common in patients with a history of myelitis and usually occur early in the course of the disease (29). In our study, sphincter function tended to significantly improve earlier in patients receiving tocilizumab, compared with that of patients receiving corticosteroids, especially in the first 6 months. The sequela was notably less severe after the 1-year follow-up.

Our results show the efficacy of tocilizumab in reducing disability is significant especially in AQP4-ab (+) NMOSD patients. However, we did not find superiority of tocilizumab over prednisone in AQP4-ab (-) patients. One possible explanation is that the effect of IL-6 receptor signaling shows different between seropositive NMOSD *versus* seronegative NMOSD. Besides, the small number of AQP4-ab (-) patients may result in the bias in comparing the efficacy between tocilizumab and prednisone.

A few studies showed a decrease in ARR following low-dose steroid therapy (30, 31), as was observed in the present study. However, treatment with steroids resulted in a higher ARR than treatment with tocilizumab. Although fewer patients receiving tocilizumab experienced further relapses, the severity of disability due to new attacks was clearly milder that that experience by patients receiving steroids. This advantage of tocilizumab over steroids requires long-term follow-ups with more patients.

There are several limitations to this study. First, the sample size was small and the study only included Chinese patients. Second, the data was obtained from a retrospective design and prospective studies will be needed to provide solid evidence of early usage of tocilizumab in acute attacks of NMOSD. Third, the pain NRS score failed to differentiate between types of pain. For example, pain localized on the skin and painful tonic spasms may be affected by segmental myelitis. Different types of pain could also influence the quality of life in various ways. Finally, objective markers such as AQP4-IgG titers, spinal cord volume, and neurofilament were not measured. The extent to which tocilizumab would affect these variables warrants further prospective study.

In conclusion, our study showed that tocilizumab treatment can effectively be initiated during acute attacks of NMOSD and can be followed by regular infusions. This strategy may accelerate improvements in disability and reduce the relapse rate, thereby providing new insights into treatment options for NMOSD.

## Data Availability Statement

The original contributions presented in the study are included in the article/**Supplementary Material**. Further inquiries can be directed to the corresponding author.

## Ethics Statement

The studies involving human participants were reviewed and approved by the Institutional Review Board of Tianjin Medical University General Hospital. The patients/participants provided their written informed consent to participate in this study.

## Author Contributions

CZ and F-DS designed the study, interpreted the data, and revised the manuscript. T-XZ and DJ provided technical supports for the study. CD, PZ and J-RH did statistical analysis. CD, PZ, J-RH, T-XZ, DJ and CZ collected and analyzed the data and revised the manuscript critically for intellectual content. Authors involved in drafting the text and figures were: CZ and CD All authors agreed to be accountable for all aspects of the work in ensuring that questions related to the accuracy or integrity of any part of the work are appropriately investigated and resolved. All authors approved the final version of the manuscript.

## Funding

The study was supported by grants from the Natural Science Foundation of Tianjin Province (18JCQNJC13200 and 20JCJQJC00280) and National Natural Science Foundation of China (81601019 and 81800853).

## Conflict of Interest

The authors declare that the research was conducted in the absence of any commercial or financial relationships that could be construed as a potential conflict of interest.

## Publisher’s Note

All claims expressed in this article are solely those of the authors and do not necessarily represent those of their affiliated organizations, or those of the publisher, the editors and the reviewers. Any product that may be evaluated in this article, or claim that may be made by its manufacturer, is not guaranteed or endorsed by the publisher.
