# Supplementary material for: Early Initiation of Tocilizumab Treatment Against Moderate-to-Severe Myelitis in Neuromyelitis Optica Spectrum Disorder
Source: Front Immunol. 2021 Oct 21;12:660230. doi: 10.3389/fimmu.2021.660230 (PMC8566740; doi:10.3389/fimmu.2021.660230)
Supplement: Supplementary file 1 [file DataSheet_1.docx]

**Supplementary**

Number of tables: 5

Number of figures: 3

**s-Table 1. Clinical scales parameters measured at acute phase and follow-ups in AQP4-ab positive NMOSD patients.**

|  | acute phase | 1-month | 3-month | 6-month | 12-month | *p* value |
| --- | --- | --- | --- | --- | --- | --- |
| EDSS | 7.0 (3.4-7.5) | 6.0 (2.5-6.5) | 3.3 (2.0-5.1) | 2.3 (2-3.6) | 2.0 (1.8-3.1) | <0.001 |
| HAI | 6.5 (1.0-8.0) | 5.5 (0.8-6.5) | 4.0 (0.0-5.0) | 2.0 (0.0-4.0) | 1.0 (0.0-3.3) | <0.001 |
| mRS | 4.0 (0.0-5.0) | 3.5 (0.0-4.0) | 2.0 (0.0-3.0) | 1.0 (0.0-2.0) | 0.5 (0.0-2.0) | 0.001 |
| Pain NRS | 6.0 (4.3-7.0) | 5.5 (3.5-6.3) | 5.0 (2.5-5.0) | 4.0 (2.3-5.0) | 4.0 (1.5-5.0) | <0.001 |
| FACIT-F | 39.0 (18.5-46.0) | 29.0 (14.8-34.8) | 25.5 (11.5-32.3) | 20.5 (7.0-24.3) | 14.0 (7.0-19.5) | <0.001 |
| ADL | 35.0 (25.0-95.0) | 75.0 (52.5-95.0) | 90.0 (78.8-100.0) | 95.0 (85.0-100.0) | 95.0 (85.0-100.0) | <0.001 |
| EQ-5D-3L | 0.4 (0.2-0.8) | 0.5 (0.3-0.8) | 0.6 (0.5-0.8) | 0.8 (0.7-0.9) | 0.8 (0.8-1.0) | <0.001 |
| Sensory function | 2.0 (2.0-3.0) | 2.0 (2.0-3.0) | 2.0 (2.0-2.0) | 2.0 (2.0-2.0) | 2.0 (2.0-2.0) | 0.003 |
| Bowel and bladder function | 1.0 (0.8-2.3) | 1.0 (0.8-2.0) | 1.0 (0.8-2.0) | 1.0 (0.8-2.0) | 1.0 (0.8-2.0) | 0.112 |

Data were shown as median (IQR).

**s-Table 2. Clinical scales parameters measured at acute phase and follow-ups in AQP4-ab negative NMOSD patients.**

Data were shown as median (IQR).

|  | acute phase | 1-month | 3-month | 6-month | 12-month | *p* value |
| --- | --- | --- | --- | --- | --- | --- |
| EDSS | 8.0 (5.8-8.0) | 7.0 (4.8-7.5) | 5.0 (2.5-6.0) | 2.5 (2.0-5.5) | 2.5 (2.0-5.3) | <0.001 |
| HAI | 8.0 (4.5-9.0) | 7.0 (3.5-7.5) | 4.0 (1.5-5.0) | 3.0 (1.0-3.5) | 3.0 (0.5-3.0) | 0.005 |
| mRS | 4.0 (2.0-4.5) | 3.0 (1.5-4.0) | 2.0 (0.5-3.0) | 2.0 (0.5-2.5) | 1.0 (0.5-2.5) | 0.053 |
| Pain NRS | 6.0 (2.5-8.0) | 5.0 (2.0-7.5) | 4.0 (1.5-7.0) | 4.0 (1.5-7.0) | 4.0 (1.5-7.0) | 0.022 |
| FACIT-F | 45.0 (21.5-47.0) | 35.0 (16.0-37.5) | 24.0 (5.5-31.0) | 21 (5.5-30.5) | 17.0 (5.5-30.5) | 0.041 |
| ADL | 20.0 (17.5-57.5) | 60.0 (37.5-80.0) | 75.0 (60.6-90.0) | 80.0 (62.5-94.5) | 80.0 (62.5-95.0) | 0.007 |
| EQ-5D-3L | 0.2 (0.1-0.5) | 0.5 (0.3-0.7) | 0,7 (0.4-0.8) | 0.7 (0.5-0.9) | 0.8 (0.5-0.9) | <0.001 |
| Sensory function | 3.0 (1.5-3.0) | 2.0 (1.0-3.0) | 2.0 (1.0-2.0) | 2.0 (1.0-2.0) | 2.0 (1.0-2.0) | <0.001 |
| Bowel and bladder function | 2.0 (0.5-3.0) | 2.0 (0.5-3.0) | 2.0 (0.5-2.0) | 2.0 (0.5-2.0) | 2.0 (0.5-2.0) | 0.094 |

**s-Table 3. Comparisons of the outcome index between patients with AQP4-ab (+) and AQP4-ab (-)**

|  | AQP4-ab (+) (n=27) | |  | AQP4-ab (-) (n=14) | | *p* value |
| --- | --- | --- | --- | --- | --- | --- |
|  | acute attack | 12-month |  | acute attack | 12-month |  |
| EDSS | 7.0 (3.4-7.5) | 2.0 (1.8-3.1) |  | 8.0 (5.8-8.0) | 2.5 (2.0-5.3) | 0.650 |
| HAI | 6.5 (1.0-8.0) | 1.0 (0.0-3.3) |  | 8.0 (4.5-9.0) | 3.0 (0.5-3.0) | 0.711 |
| mRS | 4.0 (0.0-5.0) | 0.5 (0.0-2.0) |  | 4.0 (2.0-4.5) | 1.0 (0.5-2.5) | 0.773 |
| Pain NRS | 6.0 (4.3-7.0) | 4.0 (1.5-5.0) |  | 6.0 (2.5-8.0) | 4.0 (1.5-7.0) | 0.100 |
| FACIT-F | 39.0 (18.5-46.0) | 14.0 (7.0-19.5) |  | 45.0 (21.5-47.0) | 17.0 (5.5-30.5) | 0.536 |
| ADL | 35.0 (25.0-95.0) | 95.0 (85.0-100.0) |  | 20.0 (17.5-57.5) | 80.0 (62.5-95.0) | 0.902 |
| EQ-5D-3L | 0.4 (0.2-0.8) | 0.8 (0.8-1.0) |  | 0.2 (0.1-0.5) | 0.8 (0.5-0.9) | 0.650 |
| Sensory function | 2.0 (2.0-3.0) | 2.0 (2.0-2.0) |  | 3.0 (1.5-3.0) | 2.0 (1.0-2.0) | 0.299 |
| Bowel and bladder function | 1.0 (0.8-2.3) | 1.0 (0.8-2.0) |  | 2.0 (0.5-3.0) | 2.0 (0.5-2.0) | 0.902 |

*p* values indicate the differences at 12-month between AQP4-ab (+) and AQP4-ab (-) NMOSD patients. Data were shown as median (IQR).

**s-Table 4. Comparison of the efficacy in patients with tocilizumab and prednisone at 12-month follow-up**

|  | AQP4-ab (+) | | |  | AQP4-ab (-) | | |
| --- | --- | --- | --- | --- | --- | --- | --- |
|  | Tocilizumab (n=12) | Prednisone (n=14) | *p* value |  | Tocilizumab (n=7) | Prednisone (n=8) | *p* value |
| EDSS | 2.0 (1.8-3.1) | 5.5 (2.0-6.5) | 0.049 |  | 2.5 (2.0-5.3) | 2.5 (1.5-6.5) | 0.244 |
| HAI | 1.0 (0.0-3.3) | 4.5 (1.3-7.0) | 0.005 |  | 3.0 (0.5-3.0) | 2 (0.0-6.5) | 0.085 |
| mRS | 0.5 (0.0-2.0) | 2.5 (1.0-4.0) | 0.001 |  | 1.0 (0.5-2.5) | 1.5 (0.0-4.0) | 0.165 |
| Pain NRS | 4.0 (1.5-5.0) | 5.5 (4.0-6.8) | 0.003 |  | 4.0 (1.5-7.0) | 4.0 (0.0-6.3) | 0.954 |
| FACIT-F | 14.0 (7.0-19.5) | 31.5 (23.8-37.8) | 0.000 |  | 17.0 (5.5-30.5) | 16.0 (7.8-37.2) | 0.489 |
| ADL | 95 (85-100) | 72.5 (26.3-95.0) | 0.007 |  | 80.0 (62.5-95.0) | 85.0 (25.0-96.3) | 0.103 |
| EQ-5D-3L | 0.8 (0.8-1.0) | 0.5 (0.3-0.8) | 0.008 |  | 0.8 (0.5-0.9) | 0.8 (0.4-0.9) | 0.120 |
| Sensory function | 2.0 (2.0-2.0) | 2.5 (2.0-3.0) | 0.804 |  | 2.0 (1.0-2.0) | 2.0 (2.0-2.0) | 0.196 |
| Bowel and bladder function | 1.0 (0.8-2.0) | 1.0 (1.0-3.0) | 0.208 |  | 2.0 (0.5-2.0) | 0.5 (0-1.3) | 0.655 |

Data were shown as median (IQR). Inter-group covariance analysis suggested that AQP4-ab (+) patients with tocilizumab had better response in EDSS, HAI, mRS, pain NRS, FACIT-F, ADL, and EQ-5D-3L, compared with those with prednisone at 12-month follow-up. However, in AQP4-ab (-) patients, we did not find any significant differences in all disability index between the tocilizumab group and the prednisone group at 12-month follow-up.

**s-Table 5. Comparison of disease severity of new attacks between the patients with tocilizumab and with prednisone.**

Data were shown as median (IQR) for the scales before and after relapses. Data were shown as mean ± sd for mean change of the scales. *p*^*^ indicates the comparisons of the mean change of the scales between the tocilizumab group and the prednisone group.

|  | Tocilizumab (n=3) | | | |  | Prednisone (n=11) | | | | *p^*^* |
| --- | --- | --- | --- | --- | --- | --- | --- | --- | --- | --- |
|  | before new attack | after new attack | *p* | mean change |  | before new attack | after new attack | *p* | mean change |  |
| EDSS | 2.5 (2.3-4.5) | 3.0 (3.0-5.0) | 0.102 | 0.7 ± 0.3 |  | 3.0 (1.5-6.5) | 7.5 (4.3-8.7) | 0.003 | 2.4 ± 1.4 | 0.027* |
| HAI | 1.0 (0.5-3.5) | 1.0 (0.5-3.5) | 1.000 | 0 ± 0 |  | 3.0 (1-7.5) | 7.0 (1.0-9.0) | 0.039 | 0.9 ± 1.5 | 0.074 |
| mRS | 1 (0.5-2.0) | 1.0 (0.5-2.0) | 1.000 | 0 ± 0 |  | 2.0 (0.5-4.0) | 4.0 (0.5-4.5) | 0.059 | 0.5 ± 0.9 | 0.082 |
| Pain NRS | 4.0 (3.0-5.0) | 5.0 (3.5-6.0) | 0.157 | 0.7 ± 0.6 |  | 5.0 (3.0-6.0) | 5.0 (4.5-7.0) | 0.020 | 0.7 ± 0.9 | 0.893 |
| FACIT-F | 15.0 (15.0-26.0) | 33.0 (24.0-41.0) | 0.180 | 10.0 ± 9.2 |  | 27.0 (22.0-38.0) | 45.0 (30.5-49.0) | 0.003 | 10.9 ± 5.5 | 0.883 |
| ADL | 95.0 (95.0-72.5) | 95.0 (70.0-95.0) | 0.317 | 1.7 ± 2.9 |  | 75.0 (30.0-95.0) | 60.0 (17.5-95.0) | 0.042 | 9.1 ± 16.5 | 0.185 |
| EQ-5D-3L | 0.78 (0.57-0.79) | 0.42 (0.34-0.42) | 0.109 | 0.10 ± 0.01 |  | 0.69 (0.35-0.87) | 0.34 (0.17-0.78) | 0.003 | 0.19 ± 0.15 | 0.067 |
| Sensory function | 2.0 (2.0-2.0) | 3.0 (2.4-3.0) | 0.157 | 0.7 ± 0.6 |  | 2.0 (2.0-3.0) | 3.0 (3.0-4.0) | 0.006 | 1.1 ± 0.7 | 0.346 |
| Bowel/bladder function | 1.0 (1.0-1.5) | 1.0 (1.0-2.0) | 0.317 | 0.3 ± 0.6 |  | 1.0(1.0-1.5) | 1.0(0.5-2.0) | 0.023 | 0.7 ± 0.8 | 0.386 |

**s-Figure 1.**


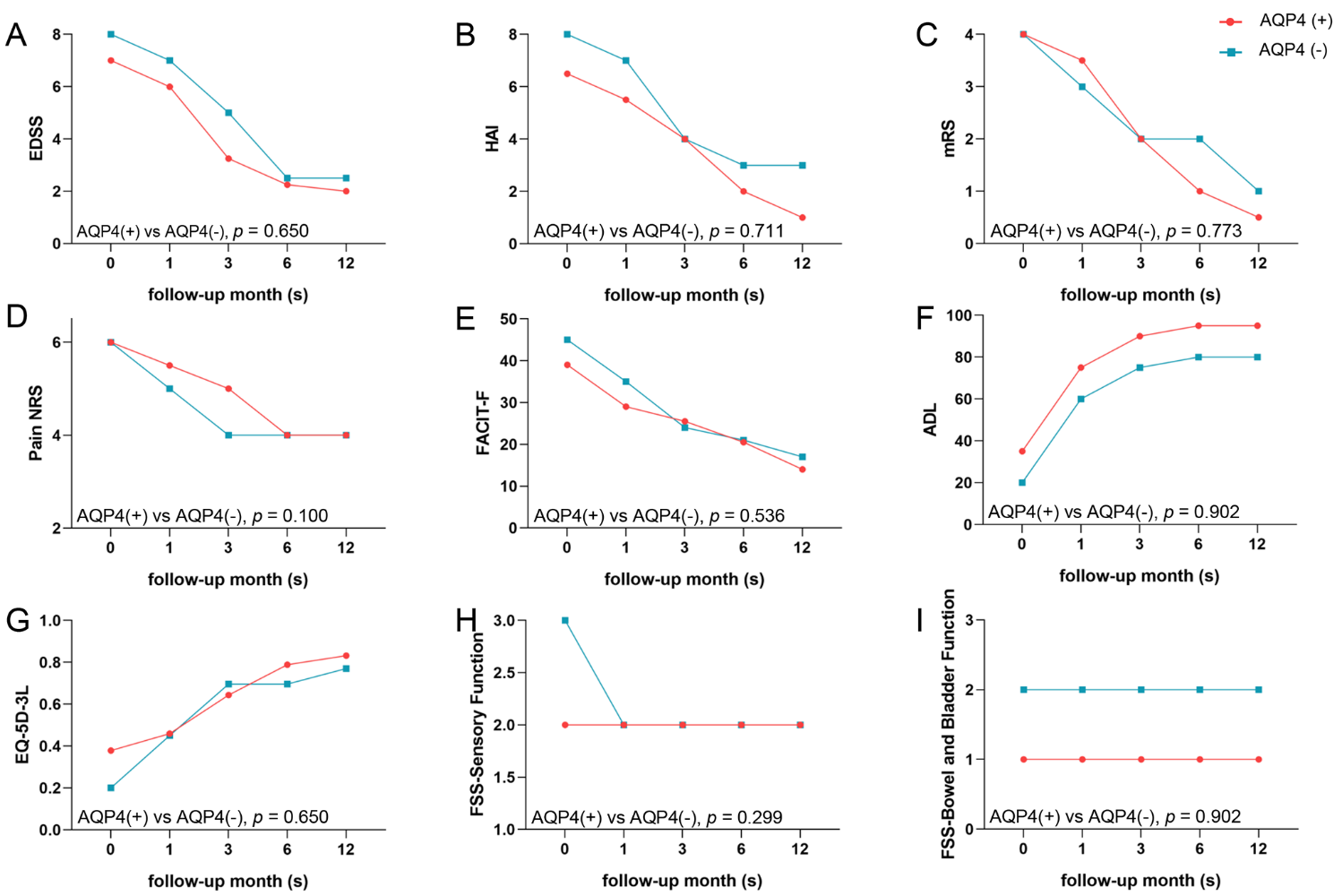


**s-Figure 1. Changes from the baseline to one-year follow-up in AQP4-ab positive and negative NMOSD patients.** EDSS (A), HAI (B), mRS (C), Pain NRS (D), FACIT-F (E), ADL (F), EQ-5D-3L (G), FSS-sensory function (H), FSS-bowel and bladder function (I) changes from the baseline to one-year follow-up in the tocilizumab and prednisone groups. Changes in AQP4-ab positive and negative group showed no significant differences (*p* > 0.05).

**s-Figure 2.**

**
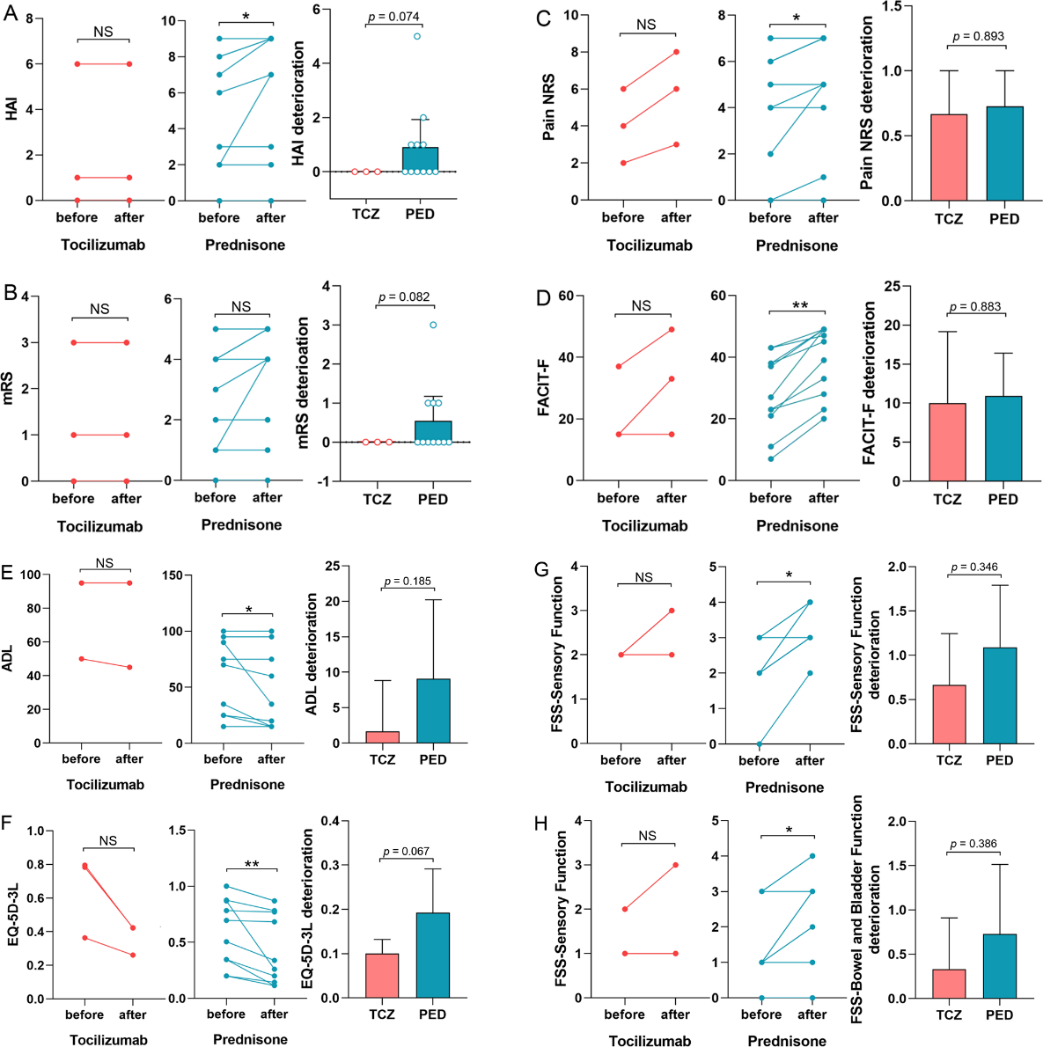
**

**s-Figure 2. Comparison of disability severity before and after new attacks in the tocilizumab group and the prednisone group.** Changes of HAI (A), mRS (B), Pain NRS (C), FACIT-F (D), ADL (E), EQ-5D-3L (F), FSS-sensory function (G), FSS-bowel and bladder function (H) changes were compared inter-group and inter-group.

**s-Figure 3.**


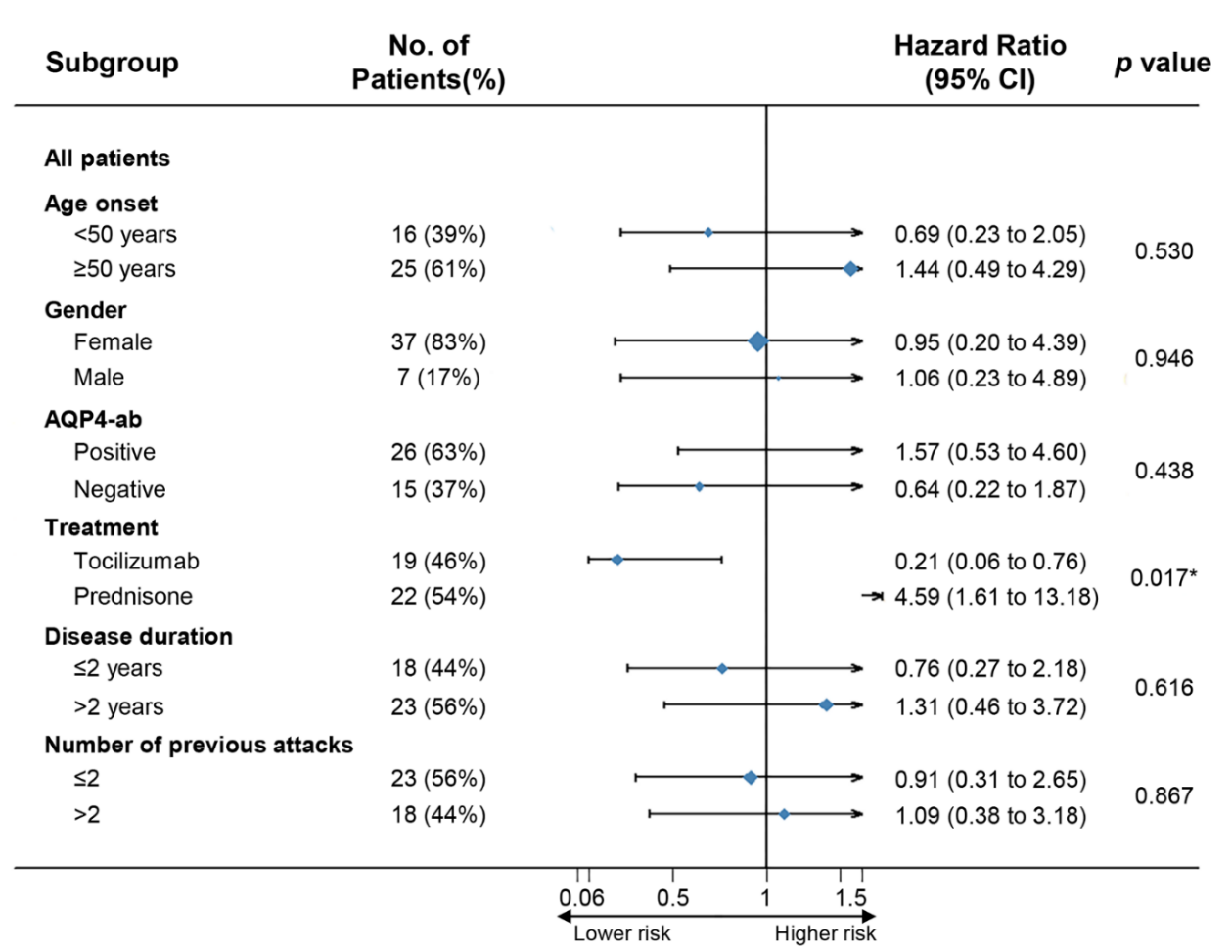


**s-Figure 3. Relapse risk in stratified analysis.** Stratified Cox proportional hazards regressions for subgroup analysis based on sex, age of onset, disease duration, presence of AQP4-ab in serum, treatment, and number of previous attacks.
